# A Case of Tetanus—Its Treatment and Cure

**Published:** 1847-11

**Authors:** T. B. Greenley

**Affiliations:** West Point, Ky.


### Art. IV.—*A Case of Tetanus—its Treatment and Cure.* By T. B. Greenley, M.D., of West Point, Ky.

Tetanus, in this climate, is of rare occurrence, especially that form called idiopathic; and when it does occur, unless it be a well developed case, it is liable to be mistaken for some other disease, such as cramp, spasm, etc., and treated accordingly. But when the symptoms manifest themselves fully, they are of so alarming a character that medical advice is generally soon sought by the friends of the patient.

The case I am about to relate is the only one that has occurred in my practice, and from the general account of authors of the fatal tendency of the disease, my apprehensions were somewhat excited as to the result. The subject was a girl about fifteen years of age, who had previously enjoyed good health, well grown, and possessing a good constitution. I was called to see her on the 20th of August, and learned the following history of the case: She was taken five days before with pains in her back, head, neck and thighs, accompanied with fever. These pains were much greater at times, when, to use the language of her mother, she acted as if she had light fits. Her head was thrown backwards from the first, and she complained of the pain being the most severe in the back part of her head and neck; bowels inclined to be constipated.

In endeavoring to ascertain the cause of her condition, I learned that on the day before she was taken sick, she waded a creek when she was very warm and perspiring freely from exercise, the weather being excessively hot. I also noticed that holes had recently been made in her ears, and lead wires bent into the shape of rings introduced. These by my directions were removed. The wading in the water under the circumstances, together with the presence of lead wires in her ears were the only assignable causes I was able to discover which would have any bearing in the production of the disease. I do not know that the lead wires would exert any deleterious influence upon the system under ordinary circumstances, but in this case I was of opinion that they might have done so, from the fact that the sterno-cleido-mastoid muscles were greatly contracted. The main cause, however, I presume, was the wading in the cold water while she was perspiring profusely, thus suddenly checking the action of the skin, and producing a considerable shock to the nervous system.

On my arrival I found her about as follows: Pulse 120, strong and tolerably full; skin hot and dry; tongue somewhat loaded, and slightly red at the tip and edges; bowels open from effects of medicine; complains of double vision, which is also slightly crossed; great contraction of the muscles of the back and neck exists, causing her head to be thrown entirely backwards, constituting well marked opisthotonos; severe pains from the back part of her head down to the sacrum, also at the pit of the stomach, and round the margins of the ribs; sterno-cleido-mastoid muscles greatly contracted, becoming powerful extensors of the head; breathing greatly oppressed, from the compression of the chest by the contraction of the thoracic muscles, together with the diaphragm; hearing somewhat indistinct; function of the kidneys appears to be healthy; menstrual discharge present. The above symptoms appeared to be permanent. At short intervals there are, in addition to these, slight spasms, at which times the pain is greatly increased, as well as the difficulty of breathing; she frequently cries out with pain, and gasps for breath.

A physician of the neighborhood was called to her the day after she was taken, and had seen her several times; but her father becoming dissatisfied with him, perhaps without reason, dismissed him, and sent for me. I did not get to see the physician, and therefore could not learn much of what had been done, nor his opinion of the case. He had cupped her on the side and temples—the latter places without scarifying. She had taken some powders, but I did not learn their composition.

*Treatment.*—The first thing I did in the way of treatment was to bleed from the arm till the surface became relaxed, and vomiting ensued. In the next place I cupped up and down the spine, taking several ounces of blood. Internally I administered free portions of Dover's powder at intervals of three hours, combined with small portions of calomel; this to be followed with a large dose of castor oil early next morning. Ordered anodyne embrocations to the sterno-mastoid muscles. Diet light but nourishing.

21st. Saw her today about ten o'clock. She became more tranquil yesterday evening after she took a second portion of the medicine; the spasms nearly disappeared, occurring at longer intervals, and much lighter; but towards morning she grew worse, and suffered much pain. The oil acted on her bowels. She complains of severe pain this morning; breathing hurried and laborious; pulse 104, but not very full; slight delirium; other symptoms about the same as yesterday.

Repeated cups to the spine. Internally, ℥ss spts. turpentine combined with ʒij laudanum, in the form of an emulsion; this to be repeated at intervals of three hours until the patient was brought under the influence of the opiate. A pint of gruel mixed with an ounce of turpentine to be administered this evening as an injection.

22d. Visited her today about the same time. Found her considerably better; slept pretty well last night, more than she has done since she was taken sick. Does not complain of much pain this morning; difficulty of breathing still exists; pulse 100, but compressible; does not talk at random as she did yesterday; has had two or three offensive discharges from the bowels since I saw her; tonic contraction of the muscles still exists. Same treatment continued.

23d. Saw her again today about the same hour. Does not seem so well as yesterday; considerable excitement of the circulation, pulse 120, and tolerably full and hard; complains of great pain in the back part of her head and neck; other symptoms about the same as yesterday.

Venesection to about sixteen ounces; free portions of Dover's powder with excess of opium at intervals of three hours, combined with small portions of calomel; injections of gruel.

24th. Visited her this afternoon. About the same as yesterday. Taken with spasms yesterday evening, which lasted nearly all night, recurring at intervals of about half an hour. These abated this morning at daybreak, and came on again about two o'clock in the afternoon.

Prescribed powders of opium, ipecac., and small portions of calomel, at intervals of three hours. Extract of belladonna to sterno-mastoid muscles. Injections of gruel. Quinine and laudanum in the morning in free portions.

25th. Saw her today about twelve o'clock. Considerably better; has had only two spasms since my last visit; head a little further forward; pulse 100, and soft.

Prescribed a dose of quinine and Dover's powder in addition to that she took this morning. Left powders of calomel and rhubarb and Dover's powder, to be taken three times daily, commencing this evening.

28th. Tetanic symptoms have nearly disappeared; has some fever; pulse 104; complains of pain in the bowels, with tenderness under pressure; discharges of a dark, grumous, bloody character, and very offensive. These symptoms indicate the presence of some irritation in the bowels, probably preceded by congestion.

Ordered a blister plaster over the umbilical region, fomentations to the abdomen, mucilaginous injections, etc.; internally, small portions of Dover's powder, quinine, and calomel.

Under this treatment the abdominal symptoms soon disappeared, and the recovery was perfect.

*Remarks.*—It will be seen that during the progress of this case, nearly all the principal remedies commonly recommended in such cases were resorted to, without effecting anything more than a palliation of the symptoms. It will also be remarked that the case assumed something of periodicity in the recurrence of the symptomps. So soon as I discovered this character, I at once resolved to try quinine and opium in free portions, and with what success the result shows. May we not from these facts conclude that malarial influences had something to do in the production of the disease in this case?

September, 1847.

